# Getting England to be more physically active: are the Public Health Responsibility Deal’s physical activity pledges the answer?

**DOI:** 10.1186/s12966-015-0264-7

**Published:** 2015-09-18

**Authors:** C. Knai, M. Petticrew, C. Scott, MA Durand, E. Eastmure, L. James, A. Mehrotra, N. Mays

**Affiliations:** Policy Innovation Research Unit, Faculty of Public Health and Policy, London School of Hygiene & Tropical Medicine, 15-17 Tavistock Place, London, WC1H 9SH UK; South Lewisham Practice, 50 Connisborough Crescent, London, SE6 2SP UK

## Abstract

**Background:**

The Public Health Responsibility Deal (RD) in England is a public-private partnership involving voluntary pledges between government, industry, and other organisations to improve public health by addressing alcohol, food, health at work, and physical activity. This paper analyses the RD physical activity (PA) pledges in terms of the evidence of their potential effectiveness, and the likelihood that they have motivated actions among organisations that would not otherwise have taken place.

**Methods:**

We systematically reviewed evidence of the effectiveness of interventions proposed in four PA pledges of the RD, namely, those on physical activity in the community; physical activity guidelines; active travel; and physical activity in the workplace. We then analysed publically available data on RD signatory organisations’ plans and progress towards achieving the physical activity pledges, and assessed the extent to which activities among organisations could be attributed to the RD.

**Results:**

Where combined with environmental approaches, interventions such as mass media campaigns to communicate the benefits of physical activity, active travel in children and adults, and workplace-related interventions could in principle be effective, if fully implemented. However, most activities proposed by each PA pledge involved providing information or enabling choice, which has limited effectiveness. Moreover, it was difficult to establish the extent of implementation of pledges within organisations, given that progress reports were mostly unavailable, and, where provided, it was difficult to ascertain their relevance to the RD pledges. Finally, 15 % of interventions listed in organisations’ delivery plans were judged to be the result of participation in the RD, meaning that most actions taken by organisations were likely already under way, regardless of the RD.

**Conclusions:**

Irrespective of the nature of a public health policy to encourage physical activity, targets need to be evidence-based, well-defined, measurable and encourage organisations to go beyond business as usual. RD physical activity targets do not adequately fulfill these criteria.

**Electronic supplementary material:**

The online version of this article (doi:10.1186/s12966-015-0264-7) contains supplementary material, which is available to authorized users.

## Introduction

Physical inactivity is a leading cause of death worldwide [1]. Globally, 6-10 % of all deaths from non-communicable diseases (NCDs) can be attributed to physical inactivity [2]. This proportion is higher for specific diseases: for example, 30 % of all deaths from ischaemic heart disease are attributed to physical inactivity [3]. In England, 26 % of women and 19 % of men are classed as inactive [4]. According to the Health Survey for England 2012, only 21 % of boys and 16 % of girls aged 5–15 met current recommendations for levels of physical activity [5]. Both boys and girls become less physically active the older they get [4].

The Public Health Responsibility Deal in England (RD) was launched in March 2011 by the Government as a public-private partnership involving voluntary agreements in the areas of food, alcohol, health at work and physical activity [6]. The RD aims to bring together those with an interest from government, academia, the corporate sector and voluntary organisations who can commit to a range of pledges which aim to improve public health. At time of writing (March 2015), 781 organisations had committed to the RD pledges [6]. Upon committing to a pledge, organisations are asked to provide a delivery plan, setting out their ideas and goals for fulfilling the pledge. Guidance is provided to organisations [7] outlining a range of interventions which they can implement. Organisations are also asked to report their progress in the spring of each year. The physical activity pledge delivery plans and progress reports are available on the RD website (https://responsibilitydeal.dh.gov.uk).

The RD is part of the Government’s current efforts to encourage physical activity in England. These include physical activity guidelines from the four UK countries’ Chief Medical Officers [8, 9]. A 2013 Public Health England briefing has also been issued on increasing physical activity and active travel in the context of reducing obesity [10], emphasising that physical activity such as brisk walking and cycling can be incorporated into everyday life and can effectively lead to weight loss [8]. The briefing underscores the importance of creating environments conducive to walking and cycling on a daily basis, as part of active travel. Encouraging and facilitating active travel is an important cross-cutting theme in current government physical activity briefings and recommendations, linking workplace health and workplace active travel schemes with local transport plans [11], supporting school travel plans [12], and employing tools such as the World Health Organization Health Economic Assessment Tool (HEAT) to conduct an economic assessment of the health benefits of walking or cycling [13], as the Department for Transport has done [14].

Though little is known about voluntary agreements to improve physical activity, lessons learned from other arenas [15–22] suggest that voluntary agreements may not lead to meaningful action in public health as they are often underpinned by an inherent conflict of interest [23, 24]. There is therefore a strong rationale for understanding the likely impact of the RD on encouraging effective actions to increase physical activity.

The RD is being evaluated in terms of its processes and its likely impact on the health of the English population, and this paper represents part of that wider evaluation, [21, 22, 25, 26] which draws on publically available data, interviews and case studies. This paper analyses the PA pledges of the RD in terms of: 1) the evidence regarding their effectiveness and; 2) the likelihood that the RD PA pledges have motivated actions among organisations that would not otherwise have taken place.

## Methods

We used distinct but complementary approaches to assess the RD PA pledges, described below. We focused our analysis on four out of the five RD PA pledges (Table 1) that had been made by the end of 2013 [6]; i.e. physical activity in the community; physical activity guidelines; active travel; and physical activity in the workplace. We did not include the pledge on physical activity inclusion (encouraging engagement with the community and communication campaigns) because it is largely covered by other PA pledges.

**Table 1:** Interventions proposed in RD physical activity pledges under analysis, and the number and proportion of interventions selected by organisations^a^, ordered by frequency of selection

Pledge (# signatories)	Interventions proposed in each pledge	Nature of the intervention^b^	Organisations listing these actions in their delivery plan	
			#	%
P1- Physical activity in the community (107 organisations)	1. Promoting community-based events locally, with campaigns targeted at specific groups within the local community (children and young people, older people or black and minority ethnic communities)	Provide information	44 (out of 107)	41%
	2. Work directly with local authorities and other local sport or physical activity providers to support or develop community-based sport and physical activity opportunities. (local sports clubs, schools, volunteer-led or other community or third sector groups).	Enable choice	25 (out of 107)	23%
	3. Sponsoring charitable events such as fun runs, cycling events, football tournaments or local walking groups	Enable choice	20 (out of 107)	19%
	4. Offer these physical activity opportunities to your employees.	Enable choice	8 (out of 107)	7%
	5. Making facilities available (at weekends).	Enable choice	0 (out of 107)	0%
P2- Physical activity guidelines (155 organisations)	6. Organisations can highlight the key messages in the CMO’s guidelines to their employees, consumers and local communities.	Provide information	96 (out of 155)	62%
	7. Organisations could also develop own materials or campaigns targeted at consumers or local communities, supported by on-pack promotions or wider associated marketing activity, for example through website or other digital media	Provide information	96 (out of 155)	62%
	8. Sign up to Change 4 Life and use their materials	Provide information	16 (out of 155)	10%
P3- Active travel (128 organisations)	9. Good quality changing, showering and locker facilities	Enable choice	52 (out of 128)	41%
	10. Providing secure, safe and accessible cycle parking	Enable choice	40 (out of 128)	31%
	11. Consider offering Bikeability training for employees to give them the confidence to cycle to work.	Provide information	43 (out of 128)	34%
	12. Sign up to the Cycle to Work Scheme	Guide choice by incentives	38 (out of 128)	30%
	13. Provision of training, reward or incentive programmes to achieve targets for more cycling.	Guide choice by incentives	28 (out of 128)	22%
	14. Provide accessible and secure cycle parking/storage or run incentive schemes to reward those who bike/walk to stores	Guide choice by incentives	25 (out of 128)	20%
	15. Encourage more of your customers to walk or cycle to your stores or sites.	Provide information	20 (out of 128)	16%
	16. Promote local walking and cycling routes to your customers, particularly those who don’t drive.	Provide information	11 (out of 128)	9%
P4- Physical activity in the workplace (203 organisations)	17. Workplace physical activity challenges	Guide choice by incentives	77 (out of 203)	38%
	18. Disseminate information on local opportunities for physical activity	Provide information	72 (out of 203)	35%
	19. Promote physical activity as part of a wider employee health and well-being programme	Enable choice	66 (out of 203)	33%
	20. Adopt policies which encourage active travel among employees	Guide choice by changing the default policy	46 (out of 203)	23%
	21. Workplace champions for physical activity	Provide information	33 (out of 203)	16%
	22. Health checks	Enable choice	21 (out of 203)	10%

Source: created by the authors. Column on pledge information drawn from Department of Health, [6]; column on “nature of the intervention” reported by authors based on the Nuffield Council of Bioethics’ Ladder of Interventions; column on “organisations listing actions” compiled by the authors

^a^as at November 2013; ^b^according to the Nuffield Council on Bioethics’ Ladder of Interventions

In November 2013, we collated all organisations’ PA pledges and delivery plans for those pledges into an Excel-based analysis framework. The framework included the names, dates of joining, delivery plan text, progress report text, individual interventions proposed in the pledge document and a summary of their ‘additionality’ (defined below). For all steps of the analysis, four researchers (CK, LJ, AM and CS) independently analysed each delivery plan or progress report, and discussed and agreed their findings in pairs.

### Types of interventions proposed within the RD PA pledges

We used the Nuffield Council on Bioethics’ Ladder of Interventions [27] to categorise the interventions within each pledge as it proposes a range of approaches to meeting public health goals, from doing nothing or providing information to consumers, to reducing or eliminating people’s choices [28].

### Analysis of signatory organisations’ pledges

Each pledge document outlines a range of possible interventions (such as provision of a “Cycle to Work” scheme) that a partner can choose to implement to deliver the pledge. We calculated the proportion of organisations selecting certain interventions (i.e. writing in their delivery plans that they would carry out a particular action, for example, engaging with local authorities to support physical activity opportunities) by dividing the number of organisations which indicated that they were planning on implementing a specific intervention by the total number of organisations which signed up to that pledge.

### Evidence synthesis

We conducted a synthesis of reviews [29] relevant to the RD PA pledges, following systematic review methods [30]. We included reviews published in any year or language which analysed the effectiveness of any relevant intervention proposed in the RD pledge documents. We included both systematic and other, less systematic reviews and categorised them as follows, according to the strength of evidence they presented: 1) Level 1 = systematic reviews, defined as a comprehensive summary of the high quality research evidence on the effectiveness of a particular intervention [31], typically involving an *a priori* comprehensive search strategy, with the goal of reducing bias by identifying, appraising, and synthesizing all relevant studies on a particular topic [32]; 2) Level 2 = reviews not meeting core criteria for systematic reviews, i.e., evidence of comprehensive search, clear selection (inclusion/exclusion) criteria and a process of quality assessment of papers reviewed. This latter group of reviews were therefore weaker methodologically, but were taken to represent “suggestive evidence”.

Reviews were included if they evaluated the effectiveness of the interventions in individuals or populations of any age group. Effectiveness was defined in terms of two outcomes: 1) increasing physical activity levels; and, 2) increasing awareness or knowledge about physical activity.

A standardised search strategy for systematic reviews was developed (included in Additional file 1) and applied to the following databases, for publications to August 2014: the Centre for Reviews and Dissemination’s Database of Abstracts of Reviews of Effects (DARE), which is the largest source of quality-assessed systematic reviews, including records of all Cochrane reviews and protocols; PubMed; and the Database of Promoting Health Effectiveness Reviews (DoPHER). We also conducted an Internet search for unpublished reviews.

Data relevant to the research questions were extracted from the selected reviews. A narrative synthesis of the data was conducted, organised by pledge. Beyond the two-fold classification described above, the quality of each review was assessed using the Measurement Tool to Assess Systematic Reviews (AMSTAR). It is an 11-item questionnaire used to rate the quality of systematic reviews by assessing the presence of an *a priori* design; duplicate study selection and data extraction; a comprehensive literature search; whether status of publication is an inclusion criteria; a list of included/excluded studies; characteristics of included studies; quality assessment of included studies; appropriate use of the scientific quality in forming conclusions; the appropriate use of methods to combine findings of studies; assessment of the likelihood of publication bias; and documentation of conflict of interest [33].

### The use of ‘additionality’ to establish the counterfactual

Conventionally, an impact evaluation seeks to establish that an intervention has caused the effects observed by using a counterfactual research design (i.e., to provide an estimate of what would have occurred without the intervention) [34]. However, attributing causality to public policies that are implemented across an entire jurisdiction can be difficult because there is no obvious comparison that can be drawn [34, 35]. The counterfactual can also be constructed qualitatively by judging so-called ‘additionality’, an approach which has been used to assess whether projects or initiatives have added value [36]. We employed the concept of additionality to help establish the counterfactual, defined as the extent to which a planned or completed activity was likely to have been brought about by the RD, as opposed to an activity which was already happening or would have happened irrespective of the RD. The counterfactual was derived from assessing organisations’ delivery plans to ascertain what actions organisations would have taken in the absence of the RD.

We developed criteria for judging the level of “additionality” in line with the Public Health Outcomes Framework’s assessment criteria for indicators [28, 37], coded from 1 to 5 (Table 2). The validation of the additionality coding scheme is reported elsewhere [22]. Statements in delivery plans and progress reports were taken at face value with no attempt to second guess organisations. This meant that our judgements, if anything, erred in favour of identifying greater additionality since there was no reason to assume that organisations would under-state their progress in relation to RD pledges.

**Table 2:** Criteria for assessing additionality

*“Have the interventions described in this delivery plan already happened, or were they going to happen regardless of the RD?”*
1. A delivery plan was coded as “1” if all interventions mentioned within were judged by assessors to be a result of the RD. Thus it was clear or very likely that the RD has motivated the partner to act by doing something new or implementing an already planned action more quickly. A fictional example is *“We will engage the community with the active travel initiative by December 2013”*
2. A delivery plan was coded as “2” if planned interventions (excluding those stated to be already completed) were judged by assessors to be potentially due to the RD. Thus the delivery plan indicated that the partner is potentially changing actions or timing of actions, or planning to, due to the RD. For example, *“We already promote a number of workplace initiatives to help encourage physical activity. We plan to add greater focus on physical activity in our marketing materials.”*
3. A delivery plan was coded as “3” if it was judged that all interventions were already implemented and/or not related to the RD. An example of a delivery plan being scored “3” would be one which stated that the signatory had already been implementing an intervention for several years prior to 2011. Thus the delivery plan clearly indicated that the partner has been doing what they describe for a while, particularly before 2011, or they have always done these activities. For example “*We have been running an active health programme for a number of years, including annual gym membership, the cycle to work scheme and showering facilities at work.”*
4. A delivery plan was coded as “4” if there was not enough information provided to make a judgement one way or the other.
5. A delivery plan was coded as “5” if no delivery plan was provided (i.e. the signatory had selected the pledge, but did not provide a plan of how to meet the pledge).

Sources: Developed by the authors and based on the Public Health Outcomes Framework [28, 37]

### Analysis of organisations’ progress on delivery of plans

We evaluated progress reports provided for PA pledges in 2014 against what had been originally set out by organisations in their delivery plans.

## Results

### What types of interventions are proposed within the RD PA pledges?

The majority of RD physical activity interventions are about provision of information to the consumer or enabling choice (Table 1).

### Who signed up to the physical activity RD pledges?

**Fig. 1:**
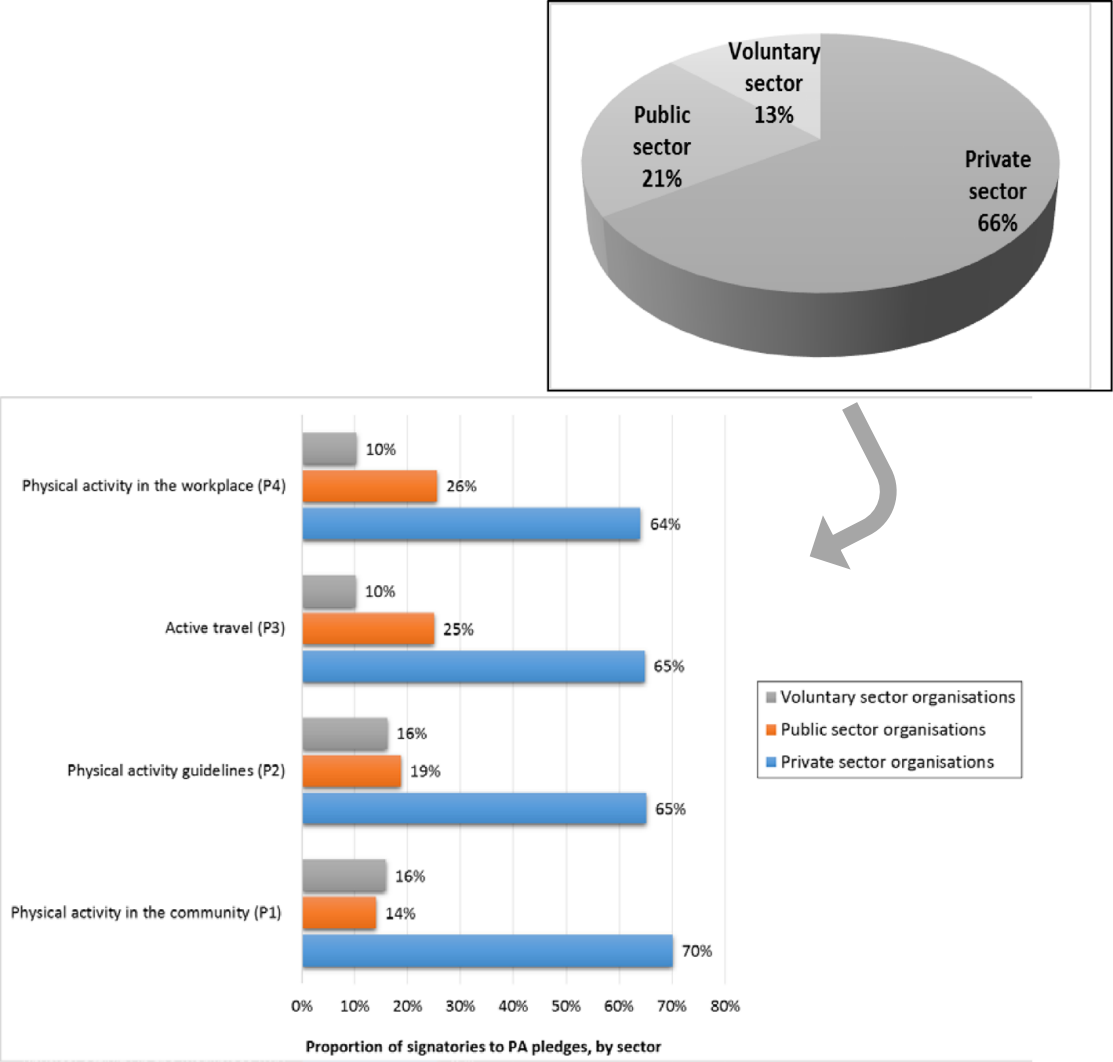
Organisations signing onto the physical activity RD pledges under analysis, by sector and by physical activity pledge

Two-thirds (66 %) of the organisations signing up to the physical activity pledges under analysis were from the private sector (including sports and fitness, food, soft drinks, alcohol, construction, energy, and health care businesses); 21 % came from the public sector (such as national governing bodies, government agencies or departments, universities and NHS trusts) and 13 % from the voluntary sector (including sports and fitness related charities, alliances and partnerships, health charities and other such organisations). There were similar ratios when disaggregated by pledge (Fig. 1). When looking more closely at which businesses signed up, 43 % of organisations which signed up to the physical activity in the community pledge were companies providing sport and fitness products or services; 23 % came from the food industry; and 4 % came from the alcoholic beverages industry. However, for the other pledges the largest category of organisations was from the food sector, followed by sport and fitness, and alcohol industries. “Charities and other voluntary organisations providing sports and fitness” was the largest category of voluntary organisation signatories.

### Which interventions did organisations list in their delivery plans?

The majority of organisations signing up to physical activity pledges committed to more than one pledge. For the four PA pledges under analysis, there were 22 different physical activity interventions (e.g. the Cycle to Work Scheme). The detail of these interventions, and the proportion of organisations selecting one of more of them in their delivery plans, are reported in Table 1.

The physical activity in the community pledge had been committed to by 107 organisations at the time of data collection at the end of 2013 (and by 102 in April 2014). The most commonly-chosen intervention under this pledge was promotion of events for specific population groups within the local community, with 41 %, or 44 organisations pledging to do so. The least preferred intervention was to make facilities available at weekends, which no organisation signed up to (0 %).

The physical activity guidelines pledge had 155 organisations signed up to it at the time of data collection at the end of 2013 (and 144 in April 2015). The most commonly-chosen interventions involved organisations highlighting the key messages from the Chief Medical Officers’ guidelines to their employees, consumers and local communities (62 %, 96 organisations); and developing materials or campaigns targeted at consumers or local communities, supported by on-pack promotions or wider associated marketing activity, for example, through website or other digital media (also 62 %, 96 organisations). The least commonly-chosen intervention within this pledge was signing up for the “Change 4 Life” campaign (www.nhs.uk/change4life) and using its materials (10 %, 16 organisations).

The active travel pledge had 128 organisations signed up to it at the time of data collection at the end of 2013 (and 138 in April 2015). Eight interventions were proposed to organisations, the most popular of which was the provision of showering, changing and locker facilities (41 %, *n* = 52). The least popular was the promotion of local walking and cycling routes to customers (9 %, *n* = 11).

The physical activity in the workplace pledge had 203 organisations at the time of data collection at the end of 2013 (and 216 in April 2015). The pledge encourages a range of interventions to organisations, the most commonly listed of which was to have workplace physical activity challenges (38 %, *n* = 77) and the least common was offering health checks to employees (10 %, *n* = 10).

### What is the evidence that these interventions will have a positive effect on physical activity?

#### Putting the RD pledges in context: the value of coordinated, complementary strategies

Overall, the evidence on strategic responses to physical activity points to the greater likely effectiveness of coordinated, multi-component, complementary approaches across the spectrum of policy interventions (compared to a reliance on isolated interventions) in and around various settings such as school [38–47] and the workplace [38, 43, 48–57]. This also includes media and educational campaigns as part of a larger multicomponent population-level strategy [38, 40, 55, 58–61], local environmental or structural changes such as improving walkability and design of neighbourhoods [38], supporting active travel initiatives for children to and from school [42–47, 56], increasing gasoline taxes to dissuade the use of cars and encourage active transport as a form of commuting [38, 62, 63], and creating safe recreation spaces [38].

#### The evidence underpinning the RD physical activity pledges

##### Description and quality assessment of reviews

**Fig. 2:**
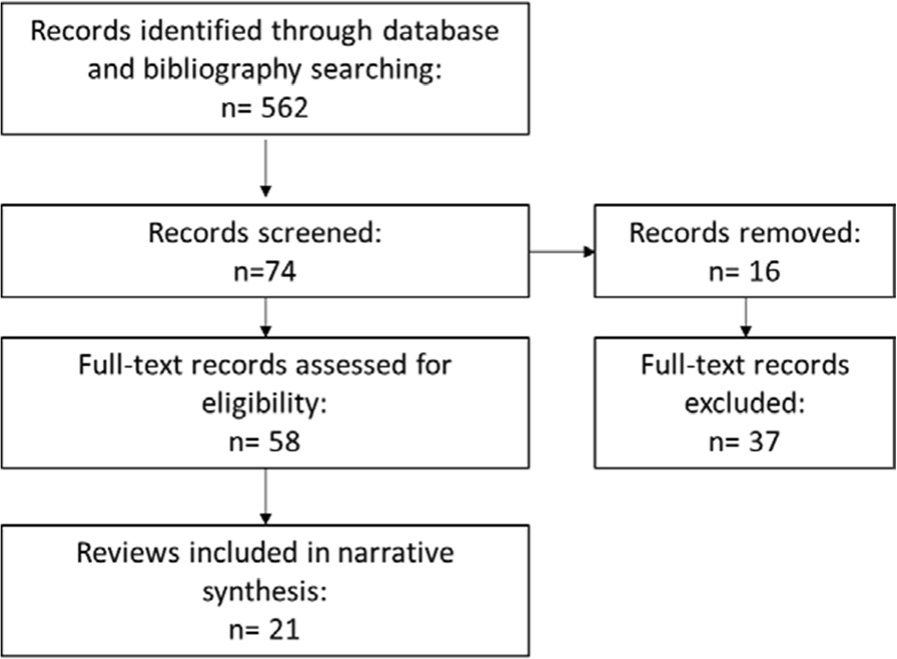
PRISMA flow chart for evidence synthesis

We identified 562 records from database searches and reference lists. After removing duplicates and screening titles and abstracts, 74 reviews were further assessed, of which 58 full text reviews were screened for potential eligibility. Twenty-one reviews published between 2007 and 2014 (17 of which were from 2010–2014 inclusive) were identified for inclusion (Fig. 2) [64]. Of the 21 reviews, 17 were systematic reviews (Level 1). The quality of reviews, assessed against AMSTAR domains, ranged from a score of 2 to 11, but with the majority (15 reviews) scoring as 8 or above on a scale of 0–11.

##### Physical activity in the community

This pledge includes media campaigns, working with local groups such as sports clubs and schools, and sponsoring charitable sporting and activity events. Twelve reviews (eight Level 1 [38, 40, 44, 55, 58, 59, 65, 66]) and four Level 2 reviews [60, 61, 65, 67] were included.

Media campaigns, the initial purpose of which is to raise the profile of physical activity within a community and to establish the relevance of physical activity to health [61], appear to achieve little in isolation but play a role if included within broader community-wide interventions: Bauman & Chau [61] updated the 2004 review by Cavill & Bauman [68] and found that the benefit of mass media campaigns is making physical activity a more visible priority for communities and decision-makers and communicating the benefits of ‘active living’ [61]. More recent reviews have reported modestly effective results [38, 55, 58], with some self-reported data suggesting improved awareness, attitudes and uptake of physical activity [69, 70]. Other reviews find inconsistent effects on physical activity [40, 59, 60]. Mass media campaigns may be effective in particular subgroups such as children with low activity levels [40]. A combination of focused media and education interventions combined with environmental approaches holds the most promise [38]. Indeed, Yang et al. [55] found that community-wide promotional activities such as motivating children and their parents to walk, and improving the built environment to favour cycling have the potential to increase cycling by modest amounts, but found that it is unclear whether increases in cycling could be achieved by addressing attitudes and perceptions about cycling. Though the RD does not specifically focus on the school setting, under the physical activity in the community pledge, organisations are encouraged to work directly with local authorities and other local sport or physical activity providers to support or develop community-based sport and physical activity opportunities, including in schools. Indeed, interventions delivered in the school setting that included physical education, activity breaks, and family involvement, appeared to be the most effective among children [40]. Moreover evidence suggests that children who have the freedom to play outdoors and travel actively in a safe environment are more physically active than those who do not [44].

To the best of our knowledge there are no systematic reviews of the literature specifically on the effectiveness of local partnerships to support physical activity. However some studies point to the importance of building on positive working history and high engagement with partners, while directly addressing issues relevant to communities involved [71, 72].

Though there is relatively little research on the direct effect on behaviour of food and drinks industry sponsorship of sporting events compared to tobacco [73–75], corporate sponsorship of sporting events is an effective form of marketing [65]. In their review, Carter et al. [66] cite two studies reporting that sponsorship of junior sport by food and beverage companies was dominated by unhealthy foods [76, 77]. Sponsorship is a way for companies to improve product and brand recall, increase the attractiveness of alcohol or low-nutrient products, and encourage purchase and consumption of those products [66]. Eight of the 22 organisations listing sponsorship of charitable events in their delivery plan were major food manufacturers and retailers.

##### Physical activity guidelines

One systematic review of randomized trials was included (Level 1 [78]. The authors reviewed the evidence (27 studies) of physical activity prescription through guidelines on behavioural adherence. Though there were methodological limitations to these studies, the authors suggest that recommended guidelines on frequency, intensity, duration and mode of activity may not have an effect on adherence, and suggested that factors unrelated to guidelines are more significant, including environmental factors, as noted above.

##### Active travel

Four systematic reviews were included [43, 47, 55, 57]. This pledge focuses on active travel among employees and customers (active travel in other population groups is discussed under the physical activity in the community pledge). Both Saunders et al. [43] and Hosking et al. [47] included studies which aimed to assess the effect of a promotional pack or advice to individuals on active travel, finding overall inconsistent results, though Kassavou et al. [57] found that interventions to promote walking in groups increase physical activity. Yang et al. [55] found that improving the infrastructure for cycling has the potential to increase cycling by modest amounts.

##### Physical activity in the workplace

Eight reviews focused on increasing physical activity opportunities in the workplace (seven Level 1 [38, 48, 50–54] and one Level 2 [49]). There were mixed results about the effectiveness of workplace physical activity interventions [51], including a Cochrane review of pedometer interventions in the workplace [52]. Wellness at work programmes including internet-based physical activity interventions [48] were more successful if they included some physical contact and environmental modification [50]. Indeed, multi-component interventions, including activities at social and environmental levels were more likely to be effective [48] and were considered the most effective at changing employee behaviour [49]. Three reviews reported positive effects of interventions at work to increase employees’ use of stairs [38, 53, 54].

### What is the likelihood that the RD encouraged action on physical activity? The “additionality” of the RD

**Fig. 3:**
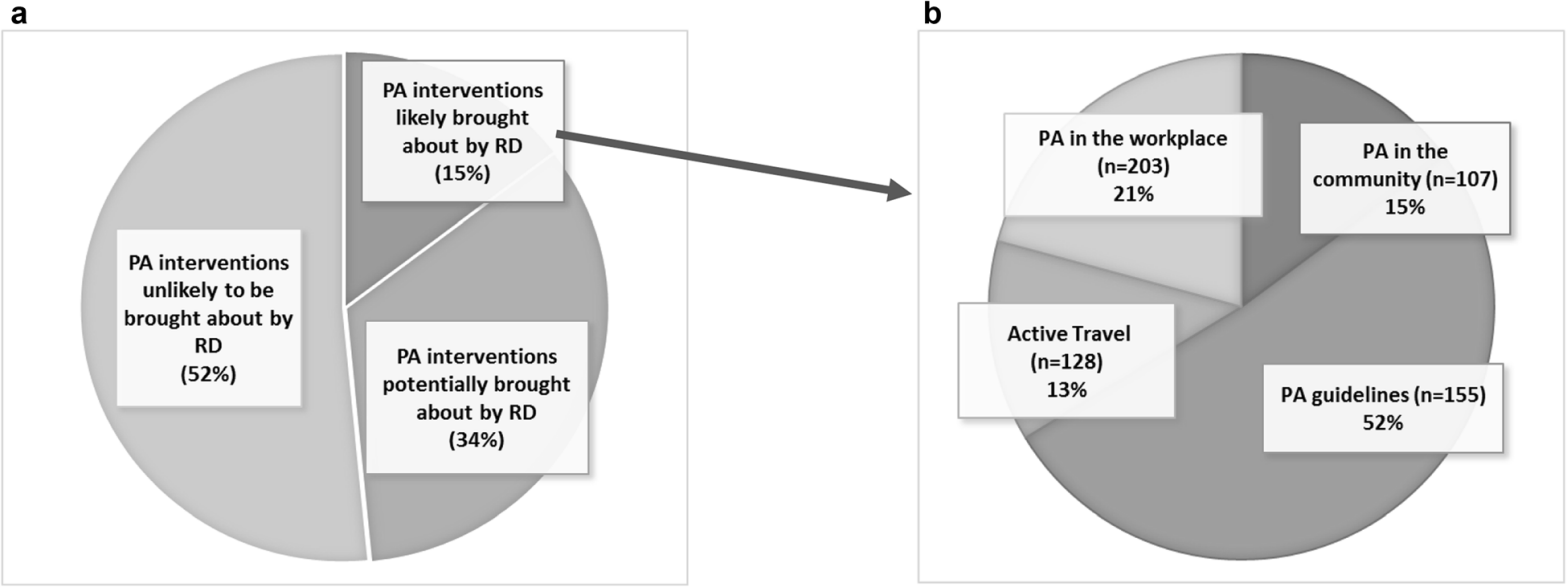
**a.** Overall proportion of interventions and whether they were likely encouraged by the RD, across the physical activity pledges. **b.** Out of 128 interventions judged to be encouraged by the RD (additionality code “1”), proportion across four physical activity pledges^a^. ^a^The number in brackets indicates the number of organisations who signed up to each pledge. So for example, the active travel pledge (P3) had a total of 128 organisations at the time of data collection, and 13% of the interventions these organisations put in their delivery plans were judged to be motivated by the RD

We counted 877 occasions in delivery plans where signatory organisations discussed either planning or having already implemented specific physical activity interventions. Of these, 128 interventions (15 %) were scored by us as likely attributable to participation in the RD. A further 297 interventions (34 %) were scored as potentially having been encouraged by the RD and 452 interventions (52 %) were assessed as either having already happened, or having been already underway when the RD started (Fig. 3) – and therefore not attributable to the RD. Of the interventions judged as having been motivated by the RD, the majority (52 %) were from the physical activity guidelines pledge.

**Fig. 4:**
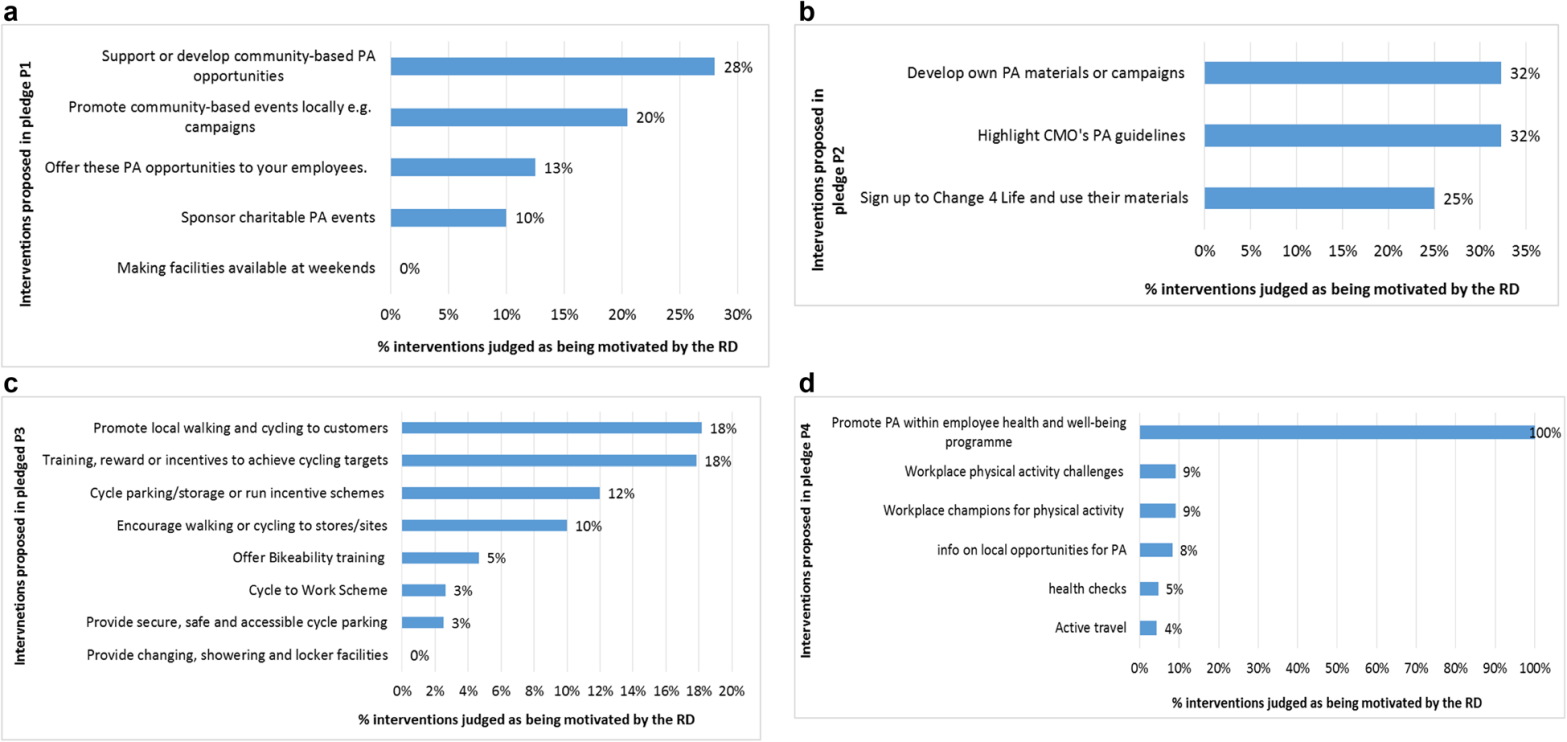
**a.** Interventions in the ‘physical activity in the community’ pledge likely brought about by the RD. **b.** Interventions related to ‘physical activity guidelines’ pledge likely brought about by the RD. **c.** Interventions in the ‘active travel’ pledge likely brought about by the RD **d.** Interventions in the physical activity in the workplace likely brought about by the RD

Within the physical activity guidelines pledge, although 62 % of organisations discussed the development of physical activity materials and campaigns and the promotion of the CMO's physical activity guidelines in their initial delivery plans, about half (32%) were judged as being due to participation in the RD (Fig. 4). However, 62 % of organisations had listed these interventions in their initial delivery plans. Within the active travel pledge, although providing changing and showering facilities was the most often selected intervention in organisations’ initial delivery plans (41 %) (Table 1), none were judged as having been motivated by the RD (Fig. 4), meaning that there is a strong likelihood that most organisations choosing this intervention would have done so regardless of the RD. Similarly, though 23 % of organisations that had signed up to the physical activity in the workplace pledge listed ‘adopting policies to encourage employees’ active travel’ as part of their plan to meet the pledge, only 4 % were judged as having been motivated by the RD to do so.

### What progress have organisations made in meeting pledges?

We assessed the progress of organisations whose actions were judged as having been motivated by the RD. This amounts to 12 out of a total 107 signatories to the physical activity in the community pledge, 32/155 signatories to the physical activity guidelines pledge, 9/128 signatories to the active travel pledge (*n* = 128), and 11/203 signatories to the physical activity in the workplace pledge. Of these, just over half (56 %) provided progress reports for 2014; 13 % provided progress reports for 2013, but not 2014; nearly one-fifth (17 %) did not provide 2013 or 2014 progress reports; and 14 % were no longer listed as partners in November 2014.

Among those reporting progress in 2013 and 2014, examples were given of interventions and the estimated reach of these actions. However, it was difficult to differentiate between interventions which had been motivated by having signed up to the RD, and actions that would have taken place anyway. For example, many sports and physical activity organisations reported providing healthy living courses or swimming lessons to their customers, but this would seem to be the focus of their usual business. Finally, there were many statements about intentions to continue to share information (e.g. physical activity guidelines), and many estimates that the majority of customers or employees had access to information about physical activity; however, these are not measures of impacts on attitudes, intentions or behaviour relating to physical activity. Therefore progress reports were not a source of usable data to evaluate whether targets were being met across the board .

## Discussion

**Fig. 5:**
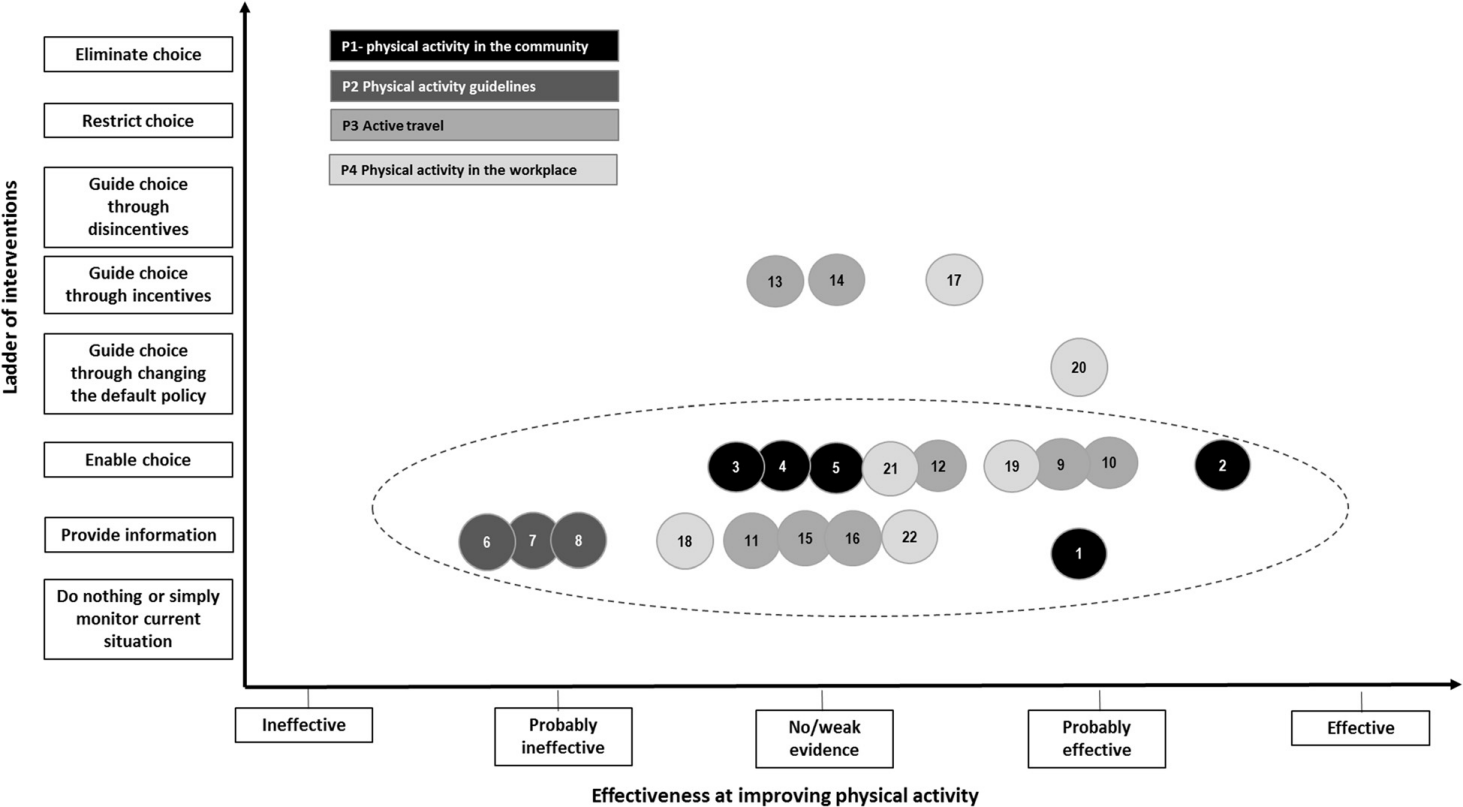
The type^a^ of interventions^b^ proposed. ^a^ Nuffield Council on Bioethics’s Ladder of Interventions [27]; ^b^ Numbers 1–22 correspond to interventions listed in Table 1

This analysis has found that the majority of RD PA pledges proposed interventions that favoured provision of information and enabling choice (Fig. 5). By contrast, the wider evidence on strategic responses to physical inactivity points to the likely greater effectiveness of making the wider environment conducive to physical activity by employing a range of policy interventions, rather than focussing on isolated interventions.

Some RD PA interventions could be effective at encouraging physical activity, such as mass media campaigns to communicate the benefits of physical activity, active travel in children and adults, and workplace-related interventions, but the reviews underscore the importance of combining these with environmental approaches to bring about behaviour change.

Though corporate sponsorship of physical activity-based events can be positive and devoid of conflicts of interest, governments as well as community physical activity or sporting event organisers should be discerning about who provides sponsorships, as this is an important marketing opportunity for industry, with demonstrated influence on behaviour change to take up the sponsor’s product rather than physical activity itself. Sponsorship of community sporting events is therefore a marketing opportunity with demonstrated influence on brand recognition and behaviour change [67, 79, 80].

Our assessment of the potential effectiveness of RD physical activity pledges is likely to represent an overestimate because we have assumed that any pledge would be implemented to a similar standard as the same interventions evaluated in the research studies reviewed. However, it is difficult to establish the level of implementation of pledges within organisations, given that progress reports were mostly unavailable, and where provided, it was often difficult to ascertain the relevance of the activities reported to the RD pledges. This made it difficult to provide systematic assessments of organisations’ progress since 2011. A review of criteria for successful voluntary agreements conducted by the authors underscores the critical importance of well-defined and independently monitored, measurable targets [26]. Any policy, whether mandatory or voluntary (such as the RD), is likely to be undermined by inconsistent self-monitored reporting systems.

Aside from the strength of the evidence underpinning the PA pledges, whether they were implemented by organisations and the quality of reporting on implementation, it is important to understand the ability of the RD, a voluntary agreement with public health goals, to motivate organisations to act differently - in this case, to implement physical activity interventions *because* of the RD. Within the active travel pledge, although providing changing and showering facilities was the most often selected action in organisations’ initial delivery plans (41 %), no examples of this intervention were judged as having been motivated by the RD. This means that there is a strong likelihood that most organisations choosing this intervention had already done so regardless of the RD. Similarly, though 23 % of signatories to the physical activity in the workplace pledge listed ‘adopting policies to encourage employees’ active travel’ as part of their plan to meet the pledge, only 4 % of organisations were judged as having been motivated by the RD to do so. There is therefore a risk that the PA component of the RD fosters a misleading perception that there have been many new efforts to address physical activity in a cross-sectoral manner when in reality signatories are largely reporting actions that they would have undertaken anyway. This is not necessarily to imply that no new actions are being undertaken in England to increase physical activity, simply that the RD’s contribution even in the case of its signatory organisations is very limited.

These findings have implications for the RD and for any similar voluntary agreements in future if they are to be more effective in contributing to better public health. First, any pledges or targets need to be well-defined, specific and measurable. The PA pledges as they are now are often vaguely formulated, and therefore difficult to evaluate. Targets also need to be measurable, relate to actions most likely to be effective in improving health (in this case, increasing levels of physical activity) and require participant organisations to go beyond ‘business as usual’. Second, progress reporting needs to be consistent and comprehensive, ideally involving some form of independent, public monitoring. Current limitations of progress reporting on delivery include that reports are all self-reported with apparently limited scrutiny of the information provided and whether it is likely to be biased towards positive reports. Finally, to maximise success, all actors participating in implementing a public health policy need to be held accountable and demonstrate progress on delivering targets [26].

### Limitations of the analysis 

Firstly, there may be unpublished or ongoing reviews we did not locate. Secondly, there are limitations to using the Ladder of Interventions as the categories are broad and may not necessarily reflect the best fit for some of the interventions described; however it helps provide an overall sense of the nature of interventions proposed in the RD PA pledges. Thirdly, the variable reporting standards were an important limitation of the evaluation, making it difficult to provide more systematic assessments of signatories’ progress. Finally, although we made every effort to validate our assessment methods, these remain a judgement of delivery plans written by organisations which may not initially have received much guidance on what and how to write their delivery plans. Thus it is possible, though highly unlikely, that organisations under-played their achievements.

## Conclusion

The RD physical activity pledges are likely to have limited effect at increasing physical activity since they are not drawn from the most effective interventions available. Implementation of RD interventions was difficult to establish because of the paucity and heterogeneity of progress reports. Moreover, only a small proportion of the actions to improve opportunities for physical activity reported by organisations signing up to the PA pledges appeared to have been motivated by the RD. Irrespective of the nature of a public health policy to encourage physical activity, targets need to be evidence-based, well-defined, measurable and push actors to go beyond ‘business as usual’.

## Additional file

Additional file 1:
**It is the Search Strategy.** (DOCX 12 kb)
