# Supplementary material for: Getting England to be more physically active: are the Public Health Responsibility Deal’s physical activity pledges the answer?
Source: Int J Behav Nutr Phys Act. 2015 Sep 18;12:107. doi: 10.1186/s12966-015-0264-7 (PMC4574469; doi:10.1186/s12966-015-0264-7)
Supplement: Additional file 1: — It is the Search Strategy. (DOCX 12 kb) [file 12966_2015_264_MOESM1_ESM.docx]

Additional file 1

**Search strategy**

| Year of pub. | No time limitations |
| --- | --- |
| TEXT / Abst / keyword | P1 Physical activity: community: (communit* OR local OR charitable OR fun run) AND (physical activity OR sport OR exercise) AND (Effectiveness OR evaluation OR assessment OR impact) AND (Health OR behaviour OR knowledge OR attitude OR perception) |
|  | P2 Physical activity guidelines: (guideline* OR standard OR advice OR recommendation) AND (physical activity OR sport OR exercise) AND (Effectiveness OR evaluation OR assessment OR impact OR...) AND (Health OR behaviour OR knowledge OR attitude OR perception) |
|  | P3 Active travel: (active AND (travel OR transportation OR mobility)) AND (physical activity OR sport OR exercise) AND (Effectiveness OR evaluation OR assessment OR impact OR...) AND (Health OR behaviour OR knowledge OR attitude OR perception) |
|  | P4- physical activity in the workplace (workplace* OR office OR worksite*) AND (physical activity OR sport OR exercise) AND (intervention OR program*) AND (Effectiveness OR evaluation OR assessment OR impact OR...) AND (Health OR behaviour OR knowledge OR attitude OR perception) |
